# Transcriptomic analysis reveals adaptive strategies to chronic low nitrogen in Tibetan wild barley

**DOI:** 10.1186/s12870-019-1668-3

**Published:** 2019-02-11

**Authors:** Xiaoyan Quan, Jianbin Zeng, Guang Chen, Guoping Zhang

**Affiliations:** 1 Agronomy Department, Institute of Crop Science, Zhejiang University, Hangzhou, 310058 People’s Republic of China; 2 School of Biological Science and Technology, University of Jinan, Jinan, 250022 People’s Republic of China; 3 College of Agronomy, Qingdao Agricultural University, Qingdao, 266109 People’s Republic of China

**Keywords:** Barley, Chronic low N stress, Low N tolerance, RNA-Seq, Genotypes, Differentially expressed genes

## Abstract

**Background:**

Development of crop cultivars with high low nitrogen (LN) tolerance or nitrogen use efficiency (NUE) is imperative for sustainable agriculture development. Tibetan wild barley is rich in genetic diversity and may provide elite genes for LN tolerance improvement. Little has been known about transcriptional responses of the wild barley to chronic LN stress.

**Results:**

In this study, transcriptomic profiling of two Tibetan wild barley genotypes, LN- tolerant XZ149 and LN-sensitive XZ56 has been conducted using RNA-Seq to reveal the genotypic difference in response to chronic LN stress. A total of 520 differentially expressed genes (DEGs) were identified in the two genotypes at 12 d after LN stress, and these DEGs could be mainly mapped to 49 metabolism pathways. Chronic LN stress lead to genotype-dependent responses, and the responsive pattern in favor of root growth and stress tolerance may be the possible mechanisms of the higher chronic LN tolerance in XZ149.

**Conclusion:**

There was a distinct difference in transcriptional profiling between the two wild barley genotypes in response to chronic LN stress. The identified new candidate genes related to LN tolerance may cast a light on the development of cultivars with LN tolerance.

**Electronic supplementary material:**

The online version of this article (10.1186/s12870-019-1668-3) contains supplementary material, which is available to authorized users.

## Background

As an essential component of key macromolecules, nitrogen (N) is quantitatively the most important mineral nutrient for plants [1]. In soils, N is often the most important factor limiting plant growth, and plants frequently encounter N deficiency in their natural habitats. In the past several decades, the increasing use of N fertilizers in crop production has played a major role in increasing yields [2]. However, the main problem is the fact that crop plants only use less than half of the applied N [3], with the remaining N causing severe environmental pollution. There is thus an impending need to realize high productivity while decreasing the rate of N application. This necessitates a comprehensive understanding of molecular mechanisms underlying morphological and physiological adaptation to low N (LN) stress in crops.

Under LN stress, the sessile plants have evolved many adaptive responses. Plants improves the efficiency of N uptake by modifying root architecture, enlarging root system and enhancing the expression of high-affinity transport systems for nitrate and ammonium [4–9]. Meanwhile, N utilization efficiency could be also improved in the plants [10]. In addition, the remobilization of N from source organs might be stimulated when plants are subjected to N limitation [11], resulting in the enhancement of N re-assimilation, and maintenance of N economy in plants [12, 13]. Furthermore, the expression levels of a number of genes associated with N metabolism in plants were altered to ensure the survival or complete their life cycle [14, 15].

Tibetan annual wild barley is regarded as one of the progenitors of cultivated barley [16, 17], possessing wider genetic diversity and generally better adaption to N deficiency in comparison with cultivated barley [18]. Some wild barley genotypes with high LN tolerance were identified, providing the elite genetic materials for improving LN tolerance of barley as well as other cereal crops [19]. Two wild barley genotypes differing dramatically in LN tolerance were used for transcriptome analysis at early stage of LN stress (6 h and 48 h) in our previous study [20]. However, plants respond to nutrient deficiency by inducing or repressing different sets of genes at special time [21]. Obviously, the mechanisms of LN tolerance in wild barley still remain to be revealed.

Although understanding of immediate responses is necessary for revealing molecular mechanisms of LN tolerance in plants, it still needs knowing the responses to long-term LN stress. In this study, we investigated genotypic differences in transcriptomic responses to chronic LN stress using the two wild barley genotypes (XZ149 and XZ56) differing in LN tolerance. The major objective of this study was to identify genes associated with the LN tolerance and to understand the molecular mechanisms underlying LN tolerance in wild barley.

## Results

### Effect of LN stress on growth performances of two wild barley genotypes

**Fig. 1:**
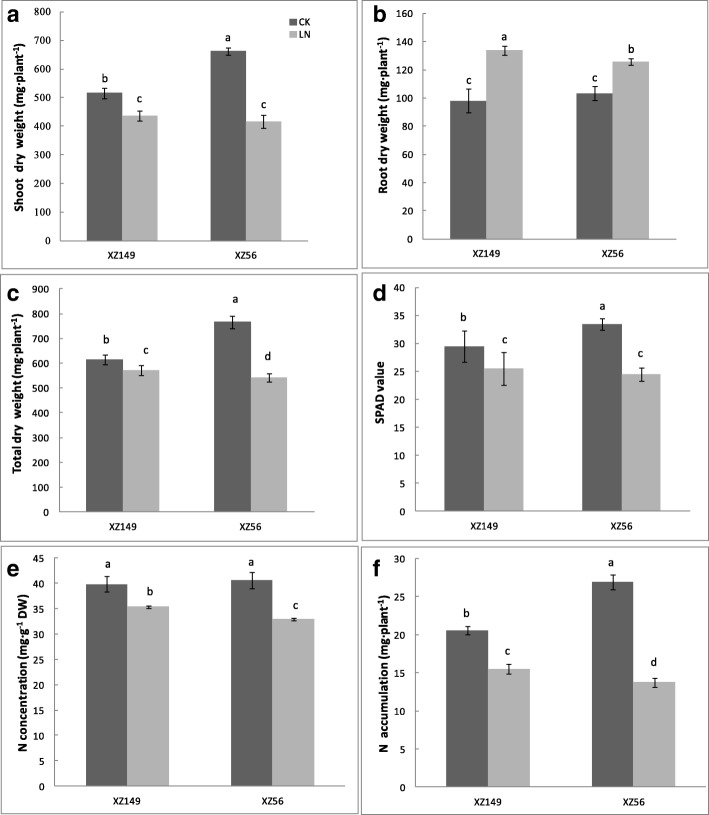
Growth performances of the two wild barley genotypes at 12 d after low N stress. **a** Shoot dry weight (*n* = 6); **b** Root dry weight (n = 6); **c** Total dry weight (n = 6); (d) SPAD value (*n* = 10); **e** N concentration (*n* = 3); **f** N accumulation (n = 6). CK: Normal N level (2 mM N); LN: Low N level (0.2 mM N); DW: dry weight. The different letters mean significant difference among treatments and genotypes according to the Duncan’s multiple range, *P* < 0.05

XZ149 and XZ56 used in this study exhibited a distinct difference in plant growth at 12 d under LN stress (Fig. 1). Shoot dry weight of the two genotypes was significantly reduced under LN stress, with 15 and 37% reduction in comparison with control for XZ149 and XZ56, respectively (Fig. 1a). On the other hand, root dry weight was increased by 37 and 22% under LN stress for XZ149 and XZ56, respectively (Fig. 1b). As a result, total dry weight per plant under LN stress was only reduced by 7% in XZ149, but as much as 29% in XZ56 (Fig. 1c). LN stress also caused a significant reduction in SPAD value (chlorophyll content), N concentration and accumulation of the two genotypes, with XZ149 being reduction of 13, 11, 25% and XZ56 of 27, 19, 49%, respectively (Fig. 1d, e and f). The less reduction of shoot dry weight, N concentration and accumulation, and SPAD value, as well as the more increase in root dry weight in XZ149 than in XZ56 proved previous finding that XZ149 is more LN tolerant than XZ56 [18].

### DEGs at the two N levels

The responding kinetics of *HvNRT2.1* to LN stress was investigated in both XZ149 and XZ56. The relative expression was obtained using pair-wise comparison between normal and LN conditions at 1 d, 2 d, 4 d, 8 d and 12 d after LN treatment, respectively (Additional file 1: Figure S1). The transcript level of *HvNRT2.1* was the maximum at 12 d after LN stress in both XZ149 and XZ56 (Additional file 1: Figure S1). In our previous study, 12 d was the time for sampling to determine the long-term changes of metabolites under LN stress [22]. Accordingly, in this study we took the samples at 12 d after LN stress for RNA-Seq analysis, to reveal the long-term responses to LN treatment.

**Fig. 2:**
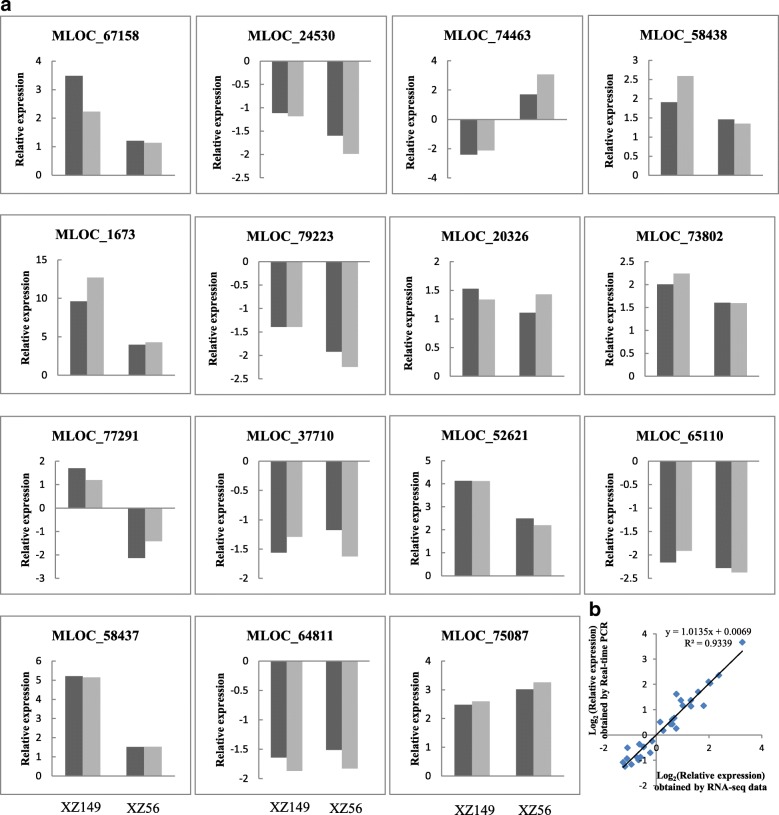
Quantitative real-time PCR validation of 15 differentially expressed genes (DEGs). **a** Transcript levels of 15 DEGs and the corresponding expression data of RNA-Seq. The grey columns represent relative expression obtained by qRT-PCR, and the black columns represent relative expression obtained by RNA-Seq. **b** Comparison between the relative expression obtained from RNA-Seq data and qRT-PCR. The RNA-Seq log_2_ value of the relative expression (y-axis) has been plotted against qRT-PCR log_2_ value of the relative expression (x-axis)

In this study, eight cDNA libraries were constructed, and a total of 132,152,200 clean reads were obtained in the all samples. For most samples, 70% of the sequenced reads could be uniquely mapped (Additional file 2: Table S2). The results from real time PCR of 15 responsive genes highly validated the RNA-Seq data (Fig. 2).

**Fig. 3:**
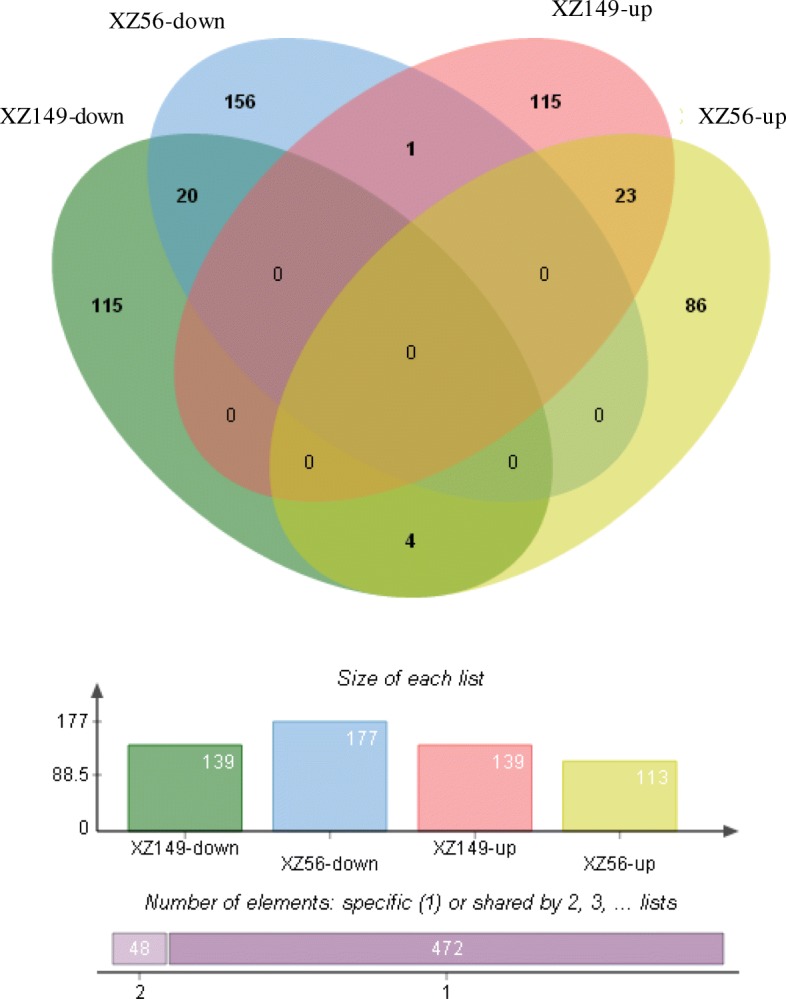
A Venn diagram describing overlaps among differentially expressed genes (DEGs) in XZ149 and XZ56

The transcriptional levels were analyzed by calculating the FPKM. Meanwhile, FPKM ≥1 at least in one of the samples and FDR < 0.05 was set as screening thresholds for DEGs. Consequently, 520 DEGs were identified using pair-wise comparison for each accession (Additional file 3: Table S3, all the sequences of the DEGs were listed in Additional file 4: Table S4), including both up-regulated (229) and down-regulated (296) genes (Fig. 3). Notably, DEGs in XZ149 (278) were almost equal to those in XZ56 (290) (Fig. 3). Meanwhile, there were 48 DEGs shared commonly in XZ149 and XZ56 (Additional file 5: Table S5). The two accessions showed the different expression patterns, with XZ149 being basically equal in up-regulated and down-regulated DEGs and XZ56 having less up-regulated DEGs than down-regulated ones (Fig. 3).

### DEGs over the different time under LN stress

Combined with the previous data on the samples taken in 6 h and 48 h after treatment, the expression patterns of different DEGs were clustered into 13 classes (Additional file 6: Figure S2). Across three time points examined, most of DGEs were only transiently down- or up- expressed at a specific time point in each genotype (Additional file 6: Figure S2). Only 29 DEGs in XZ149 and 11 in XZ56 were consistently expressed at three time points, with only one DEG being consistently up-regulated in XZ149, which encoded aminocyclopropane-1-carboxylate oxidase (ACO) (Additional file 7: Table S6). No gene was consistently down-regulated and only two DEGs, encoding nitrate transporter, were consistently up-regulated in both XZ149 and XZ56 at three time points (Additional file 7: Table S6). The results suggest that plants respond to LN stress by taking different countermeasures, inducing or repressing different sets of genes at certain time. In other words, the molecular mechanisms of LN tolerance could vary with growth stages.

### Functional classification of DEGs

**Fig. 4:**
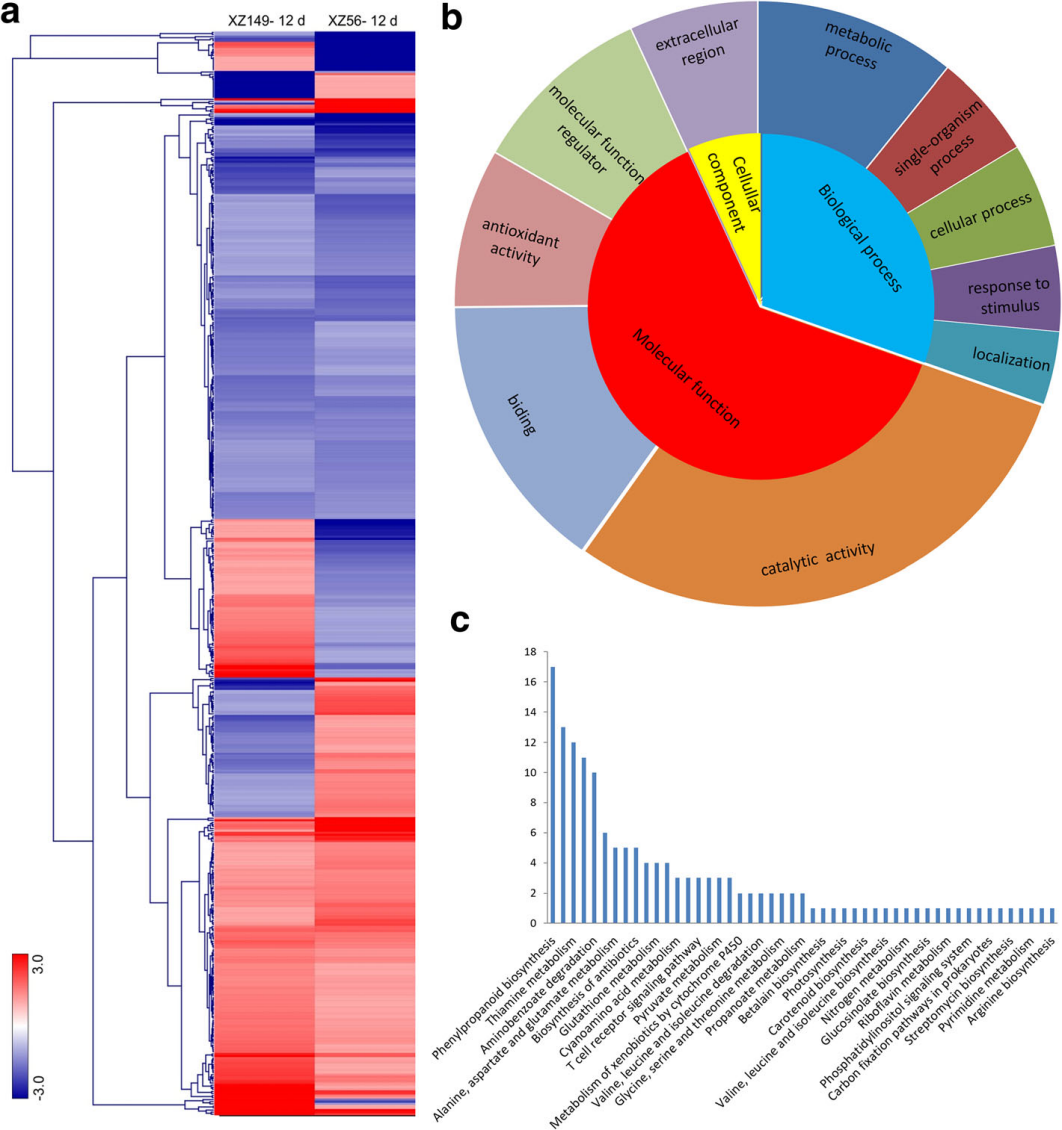
Hierarchical cluster, gene ontology (GO) enrichment and KEGG analysis of 520 DEGs at 12 d after low N stress. A total of 520 DEGs were performed on **a** Hierarchical cluster analysis. The samples and treatments are displayed above each column. Genes are displayed by different colors and relative levels of expression are showed by a color gradient from low (blue) to high (red). **b** GO enrichment. **c** KEGG overview. X- axis depicts the different pathway; Y- axis represents the number of DEGs involving in each pathway

By hierarchical clustering analysis, 520 DEGs could be clarified into four classes (Fig. 4). Furthermore, these DEGs were enriched in 10 functional groups through GO enrichment analysis (Fig. 4b, Additional file 8: Figure S3). Molecular function Ontology included four GO terms ‘catalytic activity’, ‘antioxidant activity’, ‘molecular function regulator’ and ‘binding’, while cellular component Ontology only consisted of term ‘extracellular region’. The DEGs associated with ‘metabolic process’ were the most enriched and accounted for 35% of the biological process Ontology (Fig. 4b).

KEGG pathway analysis showed that 53 encoded enzymes by the 520 DEGs were mapped to 49 KEGG pathways (Fig. 4c), such as amino acid, carbohydrate, energy and secondary metabolite metabolisms. Among these pathways, 11 DEGs were involved in sucrose and starch metabolism, and three of them were up-regulated only in XZ149 and one down-regulated only in XZ56 (Additional file 9: Table S7).

### DEGs involved in nitrogen/carbon metabolism and other nutrient uptake

Many genes involved in N absorption were differentially expressed, with ten DEGs encoding nitrate transporters, which were found at 12 d after LN stress (Table 1). Most of these DEGs were up-regulated and their relative expression was higher in XZ149 than in XZ56 except MLOC_65110 and MLOC_70747 (Table 1). In addition, asparagine synthetase (ASN) DEG (MLOC_63089) involved in N assimilation was up-regulated in XZ149 but not changed in XZ56 (Table 1), and relative content of asparagine (Asn) was also higher in XZ149 than in XZ56 in both leaves and roots (Additional file 10: Table S8). Furthermore, in comparison with XZ56, XZ149 showed higher relative content of soluble protein and activities of the two key assimilating enzymes in leaves, namely NR and GS (Additional file 10: Table S8).

**Table 1:** Genes encoding protein transporters and enzymes involve in C/N metabolism showing genotypic difference expression in response to chronic low N stress

Gene ID	Log_2_(Fold change)		Description
	XZ149	XZ56	
MLOC_1673	3.27	1.99	Nitrate transporter
MLOC_3053	0.82	0.67	High affinity nitrate transporter -like
MLOC_52621	2.04	1.32	Nitrate transporter
MLOC_58437	2.38	0.60	Nitrate transporter
MLOC_58438	0.93	0.55	Nitrate transporter
MLOC_59508	0.81	0.56	Nitrate transporter
MLOC_65110	−1.11	− 1.19	Nitrate transporter
MLOC_70747		1.51	High affinity nitrate transporter
MLOC_73802	1.00	0.68	High affinity nitrate transporter -like
MLOC_75087	1.31	1.59	High affinity nitrate transporter
MLOC_66939		0.56	Peptide transporter, putative
MLOC_36386	−0.81		Amino acid transporter-like protein
MLOC_12153	0.50		Phosphate transporter pho1–2
MLOC_15580	−0.66		High affinity potassium transporter
MLOC_17989	−0.77		High affinity potassium transporter
MLOC_54601	0.63	1.37	Nodulin MtN21 /EamA-like transporter family protein
MLOC_66056		1.44	Nodulin MtN21 /EamA-like transporter family protein
MLOC_69951		−0.48	Nodulin MtN21 /EamA-like transporter family protein
MLOC_79077	1.21		Bidirectional sugar transporter sweet3a-like
MLOC_69325	0.49		GDP-mannose transporter, putative
MLOC_38368		−0.71	Sugar transporter, putative
MLOC_9961	1.86		Sugar transporter, putative
MLOC_13104	0.67		Organic cation carnitine transporter 2-like
MLOC_15718		−1.55	Tetracycline transporter protein
MLOC_21938	0.81		Urea-proton symporter dur3
MLOC_5957		0.63	ABC transporter c family member 14-like
MLOC_62487	0.56		ABC transporter G family member
MLOC_7811	−0.70		Tonoplast dicarboxylate transporter-like
MLOC_55464	0.73		Equilibrative nucleotide transporter 3-like
MLOC_81198	−0.65		Probable transporter mch1
MLOC_63089	0.57		Asparagine synthetase 1
MLOC_44080	0.91	1.59	Asparagine synthetase 2
MLOC_60412	0.51		Soluble acid invertase
MLOC_59991	0.90		Trehalose-phosphate phosphatase
MLOC_4115		−0.72	Trehalose-phosphate phosphatase
MLOC_65390		−0.54	Pectinesterase
MLOC_59629		−0.83	Pectate lyase
MLOC_55102	0.88		Pectin acetylesterase

Blank presented in the table means no significant difference in gene expression

Carbon and N metabolisms are highly interconnected [23], and LN stress caused alteration in the expression of the genes related to carbon metabolism in barley roots. In the current study, the DEG encoding soluble acid invertase (MLOC_60412) was up-regulated in XZ149, but not changed in XZ56 under LN stress (Table 1). Two trehalose-6-phosphate phosphatase (TPP) DEGs were identified, with one (MLOC_59991) being up-regulated in XZ149 and unchanged in XZ56, and the other one (MLOC_4115) being down-regulated in XZ56 and not changed in XZ149 (Table 1). Moreover, XZ149 had higher relative contents of sucrose and trehalose in comparison with XZ56 (Additional file 10: Table S8). Futhermore, the DEGs related to cell wall remodeling, including pectinesterase (PE, MLOC_65390), pectate lyase (PEL, MLOC_59629), and pectin acetylesterase (PAE, MLOC_55102), were significantly changed under LN stress. MLOC_55102 showed elevated expression in XZ149 but not changed in XZ56, and MLOC_65390 and MLOC_59629 showed the reduced expression in XZ56 but not changed in XZ149 (Table 1).

In addition to nitrate transporter, many DEGs encoding other transporters were also identified, including phosphate transporters, potassium transporters, amino acid transporters, sugar transporters and ABC transporters (Table 1), indicating that N metabolism could affect uptake of many other nutrients in barley through interactive regulation under long-term LN stress. It was also noted that all of the four DEGs encoding sugar transporters were up-regulated only in XZ149 or down-regulated only in XZ56 (Table 1).

### Transcription factors and protein kinases

**Fig. 5:**
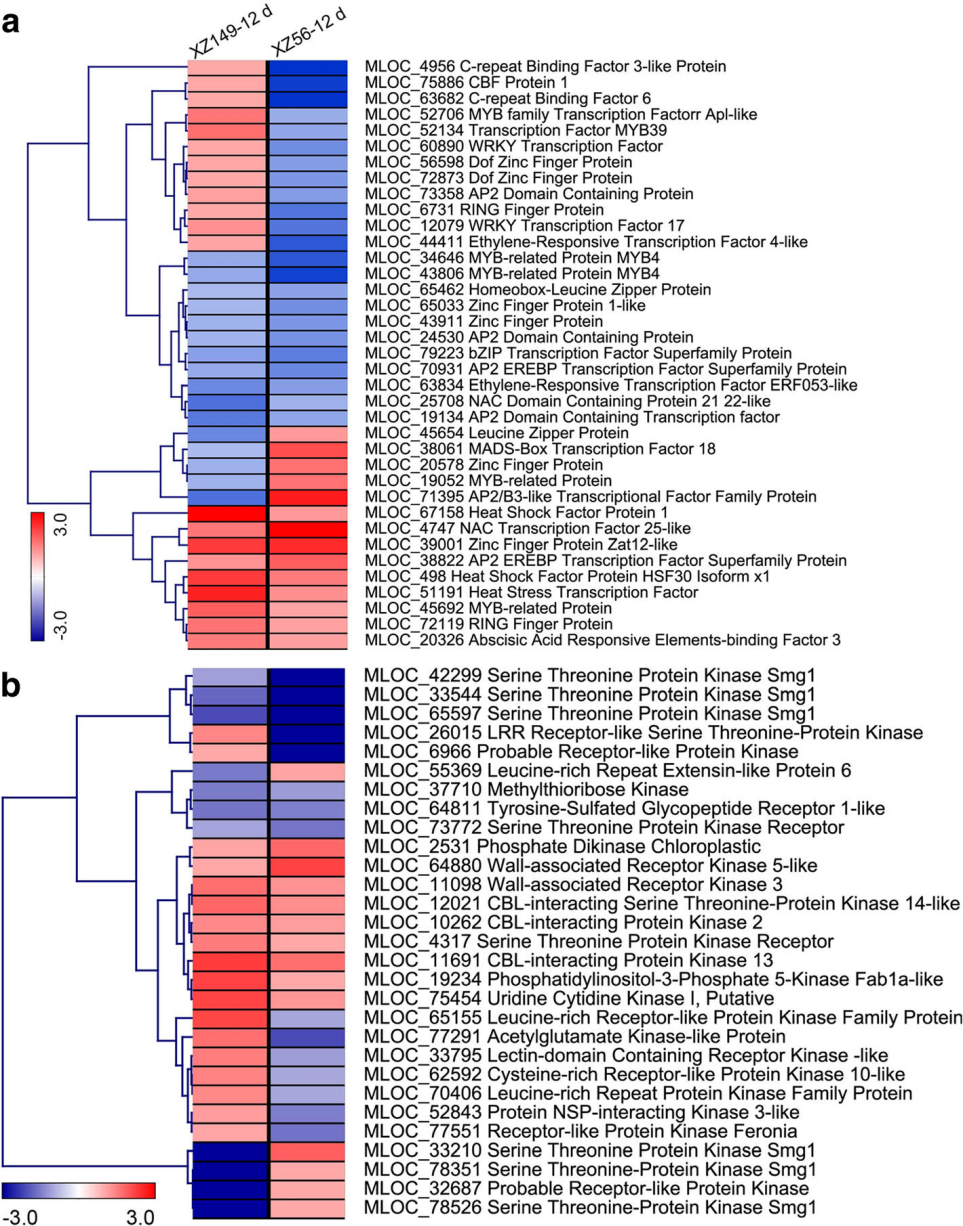
Average linkage hierarchical cluster analysis of transcription factors (TFs) and protein kinases (PKs) identified in differentially expressed genes (DEGs). **a** Transcription factors (TFs); **b** Protein kinases (PKs). The samples and treatments are displayed above each column. Genes are displayed by different colors. Relative levels of expression are showed by a color gradient from low (blue) to high (red)

Totally 37 DEGs encoding transcription factors (TFs) were identified in XZ149 and XZ56 under long-term LN stress, less than half of the early responsive TFs (89). These TFs belonged to different families, such as Zinc finger (8), ERF (8), MYB (6), HSF (3), CBF/DREB (3), bZIP (3), NAC (2), WRKY (2), MADS (1) and ABF/AREB (1) (Fig. 5a). Among the 37 TFs, only one DEG was shared by the two genotypes. All other TFs showed different responses to LN stress between the two genotypes, while most DEGs were up-regulated in XZ149 and down-regulated in XZ56. It is noteworthy that only 13 TFs were found in response to long-term LN stress in XZ149, with nine TFs being up-regulated (Fig. 5a).

In the present study, 29 DEGs encoding different groups of protein kinases (PKs) were identified under LN stress (Fig. 5b). The PKs belonging to receptor-like kinase (RLK) family was the most enriched among the PKs. Six of the fifteen RLKs carried leucine-rich repeat (LRR) domains, and three contained lectin domains (Fig. 5b). Among RLKs, a wall associated kinase 3 homolog (MLOC_11098) was up-regulated in XZ149 but not changed in XZ56 (Fig. 5b). Besides, a N-acetyl-l-glutamate kinase (NAGK, MLOC_77291) was up-regulated in XZ149 and down-regulated in XZ56, while one NSP-interacting kinase 3-like DEG (MLOC_52843) was down-regulated in XZ56 but not changed in XZ149 (Fig. 5b).

## Discussion

Nitrogen deficiency in soil is a major factor limiting crop production worldwide. It is imperative to understand the molecular mechanisms of LN tolerance in plants in order to improve LN tolerance through breeding. It is generally believed that the early responsive DEGs play more important roles for plant survival. Indeed, more DEGs were identified at 6 h (922) and 48 h (842) than at 12 d (520) after LN stress, suggesting the more genes are involved in early response to LN tolerance in plants. Moreover, plants respond to nutrient deficiency by regulating different sets of genes at special time [21]. Actually, the DEGs identified at 12 d of LN stress were different from those identified at the earlier time (Additional file 6: Figure S2). In the present study, we analyzed the transcriptomic responses of the two wild barley genotypes to LN stress by the RNA-Seq. Clearly, there is a distinct difference at the transcriptional level between the two genotypes in their responses to chronic LN stress.

### The DEGs associated directly with N metabolism

Nitrate metabolism starts from N acquisition from soil by nitrate transporters. N deficiency enhances the expression of nitrate transporters, and high affinity nitrate transporters played critical roles in N acquisition and remobilization in N-starved plants [5, 6]. The higher relative expression of most nitrate transporter DEGs in XZ149 shows its higher ability of nitrate uptake and N reallocation compared with XZ56 under LN stress, which is also confirmed by higher relative soluble protein and activity of NR and GS in the leaves of XZ149. Consequently, XZ149 had higher N concentration and more N accumulation in the plant tissues in comparison with XZ56 (Additional file 10: Table S8, Fig. 1e and f).

It is well documented that N limitation acts as an exogenous senescence-triggering factor and enhances N remobilization [11]. Amino acids from protein degradation or assimilation will be translocated and reallocated as glutamine (Gln) and Asn, the major N carriers in the phloem of many plants [24–26]. Asn synthetase gene 1 (*ASN1*), controlling Asn synthesis will be up-expressed in the plants exposed to LN stress. In addition, over-expression of *ASN1* in Arabidopsis enhanced its tolerance to limited N condition [27]. In the current study, an Arabidopsis *ASN1* homolog DEG (MLOC_63089) was only up-regulated in XZ149, but not changed in XZ56 at 12 d under LN stress. In view of higher relative content of Asn in XZ149 than in XZ56 in both leaves and roots (Additional file 10: Table S8), it may be suggested that higher LN tolerance in XZ149 is partially contributed by its better reallocation of N.

### Chronic LN stress alters carbon metabolism

It is well known that carbon and N metabolism are highly interconnected [23, 28, 29]. Indeed, 11 DEGs involved in sucrose and starch metabolism were found in barley roots under LN stress (Additional file 9: Table S7). Moreover, four DEGs encoding sugar transporters were up-regulated in XZ149 but down-regulated in XZ56 (Table 1). Meanwhile, more accumulation of sucrose was also observed in the roots of LN-treated plants relative to control (Additional file 10: Table S8), and moreover, XZ149 accumulated more sucrose in its roots than XZ56 under LN stress. As sucrose plays a role as a stimulant for lateral root formation [30], thus it could be understood that XZ149 showed better growth than XZ56 under LN stress (Fig. 1).

The gene encoding soluble acid invertase (MLOC_60412) was up-regulated in XZ149 but not changed in XZ56 after 12 d under LN stress, and the results were consistent with those in the previous study, where the sampling were taken at 6 h and 48 h after LN treatment [20]. As invertase could irreversibly catalyze the hydrolysis of sucrose [31], it may be suggested that sucrose degradation was continuously enhanced in XZ149. In fact, it is well known that invertase is crucial in the carbon supply from sucrose to the non-photosynthetic cells of plants [32]. Thus, the consistently enhanced expression of invertase in the roots of XZ149 may provide more carbon source and energy, accounting for its better root growth under LN stress.

Trehalose-6-phosphate phosphatase (TPP) involves in trehalose biosynthesis, and trehalose plays a protective role against stress in plants [33]. Currently, one DEG encoding TPP was up-regulated in XZ149 but unchanged in XZ56, and another one was down-regulated in XZ56 but not changed in XZ149. The higher relative expression of DEGs encoding TPP in XZ149 may enhance its higher biosynthesis of stress protectant trehalose (Additional file 10: Table S8). It was reported that *OsTPP1* was induced by stress [34], and its over-expression conferred stress tolerance in rice [35]. Therefore, it may be assumed that the higher relative expression of TPP and biosynthesis of trehalose in XZ149 may be a mechanism for protecting the plants against LN stress.

### The DEGs associated with cell wall remodeling

The dynamic and active structure of cell wall is necessary for plants to remodel when responding to external stimuli [36, 37]. Cell wall would be remodeled when plants are subjected to nutrition deficiency, in particular N deficiency [36]. Cell wall related genes play a role in inducing cell wall relaxation and maintaining N assimilation under N starvation [38]. In this study, we found that cell wall remodeling DEGs, including PE, PEL, and PAE were more highly expressed in XZ149 than in XZ56 under LN stress. In addition, it should be noted that above-mentioned three enzymes are involved in pectin degradation. Hence, in comparison with XZ56, more pectin breakdown in XZ149 may provide it more materials for use in other biological processes, compensating for the depressed photosynthetic carbon assimilation under LN stress [15]. Thus, higher relative expression of cell wall related DEGs in XZ149 may be beneficial for its coping with LN stress.

### Transcription factors and hormone signaling

Expression of plant-specific Dof1 TFs has been proved to improve plant growth under LN condition [39, 40]. Several R2R3-type MYB TFs are involved in plant stress responses [41], and over-expression of *OsMYB48–1* enhanced drought and salinity tolerance [42]. In this study, we found that one Dof and two MYB were down-regulated in XZ56, while three MYB were up-regulated in XZ149 (Fig. 5a). It may be assumed that these IFs may contribute to the development of complex signaling webs and should be potential candidates for improving LN stress tolerance.

The synthesis of plant hormone ethylene is tightly regulated in response to stresses [43]. Genes encoding ACO, a rate-limiting enzyme in ethylene synthesis, have been reported to be response to N deficiency [44, 45]. In our previous study, three ACO homologs were up-regulated only in XZ149 under LN stress, however all of them were transiently expressed at either 6 h or 48 h [20]. Here, another ACO DEG (MLOC_34709) was detected, which was up-regulated in XZ149 at all the three time points, but unchanged in XZ56 at the later two time points (Additional file 7: Table S6). Considering the fine tuning between NRT2.1 expression and ethylene biosynthesis under LN level [46], it may be suggested that the up-regulation of MLOC_34709 in XZ149 contributes to its early and long-term LN tolerance.

### Protein kinases

Protein kinases are important for adaptation to abiotic stress in plants [47]. The NSP-interacting kinase (NIK), belonging to the five leucine-rich repeats-containing receptor-like kinase subfamily, is a transducer of plant defense signaling [48]. In this study, we found that NSP-interacting kinase 3-like (MLOC_52843) was down-regulated only in XZ56 (Fig. 5b), suggesting that XZ149 may enhance its LN stress tolerance by maintaining its defense capability. In addition, a wall associated kinase 3 (WAK) homologous DEG (MLOC_11098) was up-regulated in XZ149 but unchanged in XZ56 (Fig. 5b). WAK is involved in root growth under LN stress [49], thus its higher expression in XZ149 could partially account for its better adaptation to LN stress.

N-acetyl-l-glutamate kinase (NAGK), the prototype of the amino acid kinase family, can catalyzes a key step of Arginine (Arg) synthesis. Arg content was increased in plants under N-deficient condition [50]. It was reported that some amino acids play roles in alleviating abiotic stress [51]. Currently, a NAGK homolog (MLOC_77291) was up-regulated in XZ149 and down-regulated in XZ56 (Fig. 5b), indicating that the increase of NAGK in XZ149 could confer its LN tolerance.

## Conclusion

The current study identified the DEGs in barley roots, which respond to chronic (12 days) LN stress. There was a dramatic difference between the two Tibetan wild barley genotypes in transcriptomic response to chronic LN stress. The pattern of genetic responses in favor of better root growth and higher stress tolerance may be the possible mechanisms of the higher LN tolerance in XZ149. In addition, some new candidate genes related to LN tolerance were identified, which could be useful for developing barley cultivars with LN tolerance.

## Methods

### Plant materials and LN treatment

The experiment using two Tibetan wild barley accessions XZ149 and XZ56 (LN- tolerant and sensitive genotypes, respectively) was carried out in black plastic pots (5 L) in a greenhouse with natural light. The wild barley accessions were collected from Tibetan area in last century and kindly presented by professor Sun of Huazhong Agricultural University, China. The nutrition solution was used according to Quan et al. [20]. The solution was renewed every five days, continuously aerated with pumps. Treatments were conducted on three-leaf-stage seedlings with two N levels (0.2 mM N as LN treatment, 2 mM N as control).

For biomass determination, the plants were harvested at 12 d after LN treatment and separated into shoots and roots. Dry weight was recorded after the samples were dried at 105 °C for 30 min and to constant weight at 80 °C. Meanwhile fresh plant tissues were taken for use in determining nitrate reductase (NR) activity, glutamine synthetase (GS) activity and soluble protein content with three biological replications, and the content of metabolites with four biological replications. The roots of the two barley accessions under both N treatments were sampled with three biological replicates at 1 d, 2 d, 4 d, 8 d and 12 d after treatments for the time course analysis of gene *HvNRT2.1* expression. For RNA-Seq analysis, the samples were taken at 12 d after treatments. Roots of four seedlings for each treatment were pooled as one biological replication. Totally eight samples [2 genotypes × 2 treatments × 2 biological replications] were taken for analysis.

### Physiological measurement

N concentration in plant tissue was determined using Foss Kjeltec 8400. Soluble protein content was measured as described by Andrews et al. [52]. NR and GS activity were determined according to Kaiser et al. [53] and Masclaux-Daubresse et al. [54], respectively. The metabolites were extracted and analyzed using 7890A/5975C GC–MS system (Agilent, USA) and AMDIS 32 software according to Quan et al. [22].

### cDNA library construction and sequencing for RNA-seq

The total RNA was extracted using miRNeasy mini kit (QIAGEN, Germany) following the manufacturer’s specification. RNA degradation, integrity, abundances and purity were assessed for meeting the requirements [20]. cDNA libraries were constructed using the Illumina TruSeq™ RNA Sample Preparation Kit (Illumina, San Diego, CA, USA) according to the manufacturer’s instructions. Briefly, mRNA was obtained from the total RNA using magnetic beads with poly-T oligonucleotide. Then the random fragmentation of the purified mRNA was reversely transcribed into cDNA. After ligated with the adapters on both ends, DNA fragments were selectively amplified and enriched. Subsequently, the purified PCR products were quantified using Agilent Bioanalyzer 2100 system. After cluster generation, the final cDNA library was sequenced on an Illumina NextSeq 500 platform.

Raw reads with 75 bp single-end were initially processed to remove adapters sequences, empty sequences and low-quality bases, and then the Q20, Q30, GC contents, and sequence duplication level of the clean data were analyzed. Then the clean reads were mapped against the barley reference genomes using TopHat (http://tophat.cbcb.umd.edu/), and finally the mapping results were analyzed to identify splice junctions.

### Identification of the DEGs and validation of RNA-Seq by real time PCR

Gene expression levels were calculated by the FPKM values (fragments per kilobase of exon per million fragments mapped reads) [55]. Fold-changes were defined as normalized read count abundance for the LN-stressed samples divided by that of the control samples. To identify differentially expression genes (DEGs), the difference in expression between control and LN treatment was analyzed using the DESeq R package (1.10.1) [56]. An FDR (false discovery rate) of 0.05 was used for determining significant DEGs [57].

To validate the reliability of the RNA-Seq results, the expression of candidate genes was determined by real time PCR assay using the RNA for RNA-Seq. The first strand cDNA was synthesized using PrimerScript™ RT reagent Kit with gDNA Eraser (Takara, Japan). The gene-specific primers, designed by primer-blast (http:/www.ncbi.nlm.nih. gov/tools/primer-blast/), were presented in Additional file 11: Table S1. All real time PCR analyses were performed on a CFX96 system (Bio-Rad USA) with two biological replicates and three technical replicates. *HvGAPDH* was used as an internal control. The relative expression was calculated by the comparative CT method and expressed as the fold change referred to the expression in the control plants [58].

### Statistical analysis

Gene Ontology (GO) annotation and KEGG (Kyoto Encyclopedia of Genes and Genomes) analysis for the DEGs were conducted using the Blast2GO program [59] according to Zeng et al. [60]. GO terms were tested by applying tools for GO enrichment (http://systemsbiology.cau.edu.cn/agriGOv2/) at *p*-values ≤0.05 [61]. Venn diagram was made on jvenn (http://jvenn.toulouse.inra.fr/app/example.html) [62]. Heatmaps and hierarchical clustering were generated with genesis 1.8.1. Significant differences of gene expression between treatments were tested using a DPS statistical software, and the difference at *P* < 0.05 was considered as significant.

## Additional files

Additional file 1: **Figure S1.** Temporal expression of the *HvHRT2.1* gene in XZ149 and XZ56 under low N stress. The relative expression was calculated by the expression of LN stress divide by that of control. Different lowercase represents significant differences according to the Duncan’s multiple range, P < 0.05, *n* = 4. Primers of *HvNRT2.1* and *GAPDH* for real time PCR are listed in Table S1. (DOCX 16 kb)

Additional file 2: **Table S2.** Summary of mapping reads of the RNA-Seq (XLSX 11 kb)

Additional file 3: **Table S3.** The FPKM value of 520 DEGs in XZ149 and XZ56 (XLSX 113 kb)

Additional file 4: **Table S4.** Gene accession numbers and sequences of 520 DEGs (XLSX 478 kb)

Additional file 5: **Table S5.** DEGs at 12 d after low N stress in XZ149 and XZ56 (XLSX 160 kb)

Additional file 6: **Figure S2.** Hierarchical cluster of DEGs at three time points in XZ149 and XZ56. The samples and treatments are displayed above each column. Genes are displayed by different colors and relative levels of expression are showed by a color gradient from low (green) to high (red). (PNG 142 kb)

Additional file 7: **Table S6.** DEGs consistently expressed at three time points under low N stress in each genotype (XLSX 13 kb)

Additional file 8: **Figure S3.** GO annotation and enrichment analysis (PNG 65 kb)

Additional file 9: **Table S7.** Enzymes and sequences in one KEGG pathway (XLSX 12 kb)

Additional file 10: **Table S8.** Traits related with N metabolism in two wild barley genotypes XZ149 and XZ56 under low and normal N levels (DOC 40 kb)

Additional file 11: **Table S1.** The primers used in real time PCR (DOC 53 kb)

## Abbreviations

ACO: Aminocyclopropane-1-carboxylate oxidase
Arg: Arginine
Asn: Asparagine
ASN: Asparagine synthetase
DEGs: Differentially expressed genes
FDR: False discovery rate
FPKM: Fragments per kilobase of exon per million fragments mapped reads
Gln: Glutamine
GO: Gene Ontology
GS: Glutamine synthetase
KEGG: Kyoto Encyclopedia of Genes and Genomes
LN: Low nitrogen
LRR: Leucine-rich repeat domain containing kinase
N: Nitrogen
NAGK: N-acetyl-l-glutamate kinase
NR: Nitrate reductase
PAE: Pectin acetylesterase
PE: Pectinesterase
PEL: Pectate lyase
PKs: Protein kinases
RLK: Receptor-like kinase
TFs: Transcription factors
TPP: Trehalose-6-phosphate phosphatase
WAK: Wall associated kinase
