# Supplementary figures and images for: Transcriptomic analysis reveals adaptive strategies to chronic low nitrogen in Tibetan wild barley
Source: BMC Plant Biol. 2019 Feb 11;19:68. doi: 10.1186/s12870-019-1668-3 (PMC6371475; doi:10.1186/s12870-019-1668-3)

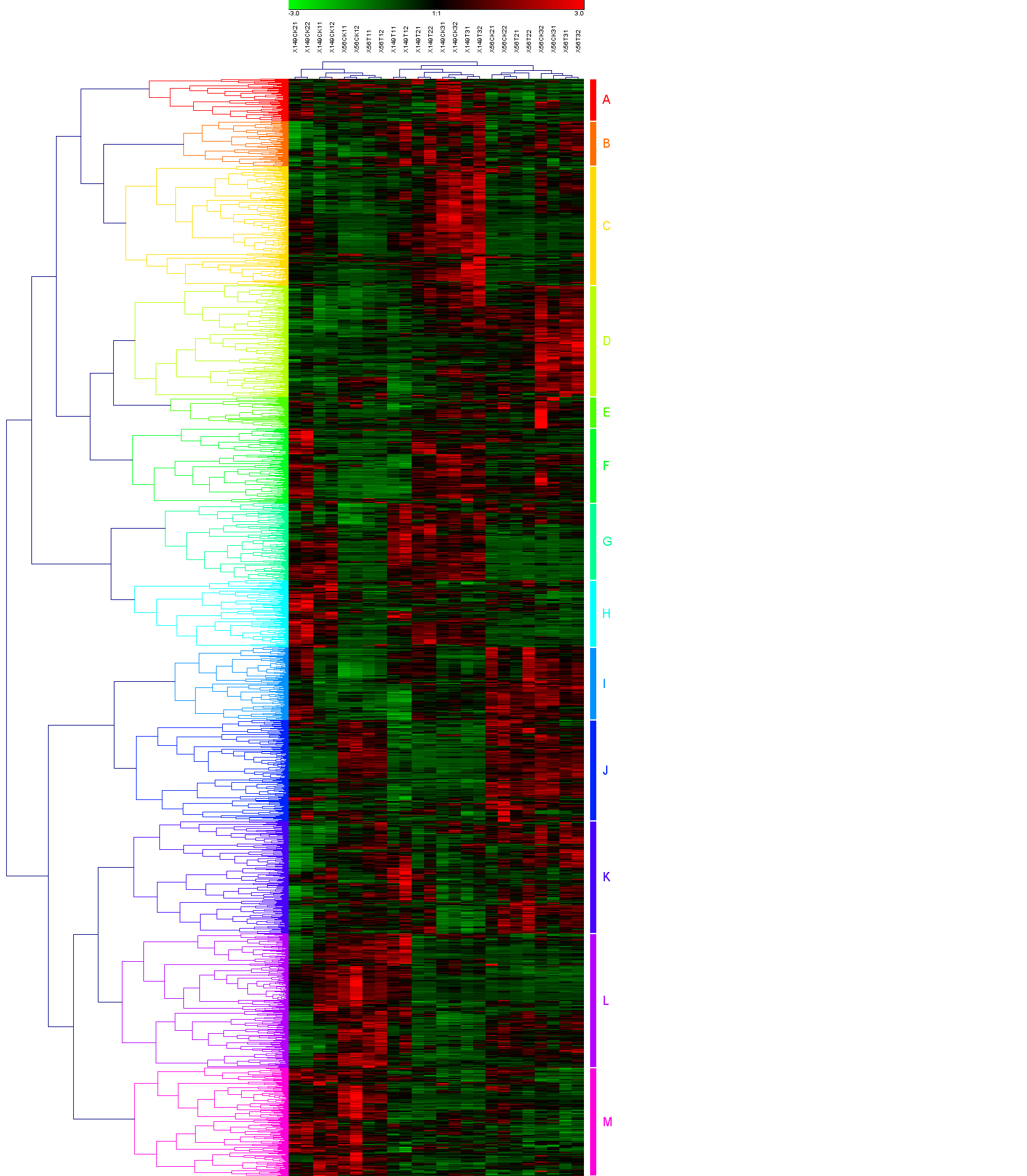

Supplement: Supplementary file 6 — Figure S2. Hierarchical cluster of DEGs at three time points in XZ149 and XZ56. The samples and treatments are displayed above each column. Genes are displayed by different colors and relative levels of expression are showed by a color gradient from low (green) to high (red). (PNG 142 kb) [file 12870_2019_1668_MOESM6_ESM.png]

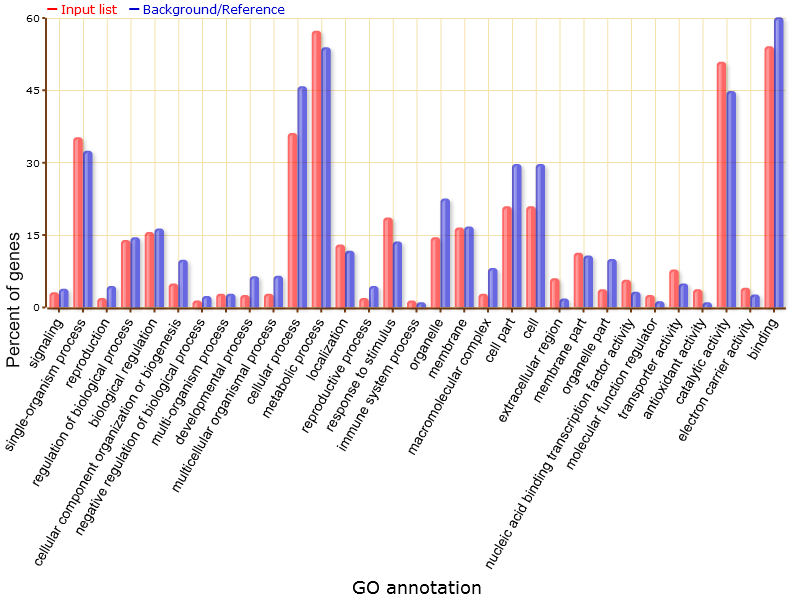

Supplement: Supplementary file 8 — Figure S3. GO annotation and enrichment analysis (PNG 65 kb) [file 12870_2019_1668_MOESM8_ESM.png]
