# Supplementary material for: Transcriptomic analysis reveals adaptive strategies to chronic low nitrogen in Tibetan wild barley
Source: BMC Plant Biol. 2019 Feb 11;19:68. doi: 10.1186/s12870-019-1668-3 (PMC6371475; doi:10.1186/s12870-019-1668-3)
Supplement: Supplementary file 10 — Table S8. Traits related with N metabolism in two wild barley genotypes XZ149 and XZ56 under low and normal N levels (DOC 40 kb) [file 12870_2019_1668_MOESM10_ESM.doc]

**Table S8. Traits related with N metabolism in two wild barley genotypes XZ149 and XZ56 under low and normal N levels**

| **Trait** |  | **XZ149** | | | **XZ56** | | |
| --- | --- | --- | --- | --- | --- | --- | --- |
|  |  | **CK** | **LN** | **Relative** | **CK** | **LN** | **Relative** |
| GS activity (U·mg^-1^ prot) | Leaf | 184.72c | 216.21a | 1.17 | 183.85c | 196.83b | 1.07 |
|  | Root | 132.93c | 320.06b | 2.41 | 135.50c | 415.30a | 3.06 |
| NR activity (mg·g^-1^·h^-1^) | Leaf | 182.75a | 17.31b | 0.09 | 141.30a | 7.94b | 0.06 |
|  | Root | 8.31a | 1.19b | 0.14 | 7.77a | 0.42b | 0.05 |
| Soluble protein content (mg·g^-1^ FW) | Leaf | 9.85b | 7.73c | 0.78 | 11.92a | 7.86c | 0.66 |
|  | Root | 3.71a | 1.94b | 0.52 | 3.38a | 1.70b | 0.50 |
| Asparagine | Leaf | 53125.14b | 86683.16a | 1.63 | 27689.33c | 13222.97d | 0.48 |
|  | Root | 25814.73b | 46420.09a | 1.80 | 20586.19c | 26375.67b | 1.28 |
| Sucrose | Leaf | 3073475.01b | 3407746.45a | 1.11 | 3125141.58b | 3446017.53a | 1.10 |
|  | Root | 1895603.24d | 2548749.72b | 1.34 | 2041126.40c | 2706234.14a | 1.32 |
| Trehalose | Leaf | 969.17c | 1608.75a | 1.66 | 931.69c | 1278.93b | 1.37 |
|  | Root | 1090.35c | 3464.03a | 3.18 | 1230.92c | 2547.86b | 2.07 |

GS: Glutamine synthetase; NR: nitrate reductase; CK: Normal N level (2 mM N); LN: Low N level (0.2 mM N); Relative: LN/CK. For each line, different lowercase letters indicate significant differences (P, 0.05) among the treatments and genotypes, n = 3. Mean metabolite content (n = 4) is reported as peak area relative to the internal standard.
