# Supplementary material for: Transcriptomic analysis reveals adaptive strategies to chronic low nitrogen in Tibetan wild barley
Source: BMC Plant Biol. 2019 Feb 11;19:68. doi: 10.1186/s12870-019-1668-3 (PMC6371475; doi:10.1186/s12870-019-1668-3)
Supplement: Supplementary file 11 — Table S1. The primers used in real time PCR (DOC 53 kb) [file 12870_2019_1668_MOESM11_ESM.doc]

| **Gene ID** | **Description** | **Primer sequence (From 5′ to 3′)** |
| --- | --- | --- |
| MLOC_75087 | High affinity nitrate transporter | F:GGTGCAATGGGGGAATACGA |
|  |  | R:GAGCACACCAAACCCAAACG |
| MLOC_73802 | High-affinity nitrate transporter -like | F:GCGTCACCACCTCCATCAAG |
|  |  | R:ACGGACGAACACCTTGTGA |
| MLOC_1673 | Nitrate transporter | F:GGTCCTCTTCTCCATCTGCG |
|  |  | R:CCCACTCAAGCCCGAAATGA |
| MLOC_52621 | Nitrate transporter | F: AGCGTGGCGTATTGCTTACT |
|  |  | R: CTTCTTCTGGAGGCTTGCGA |
| MLOC_58437 | Nitrate transporter | F:CTTTGTGGCATGGCGTATCG |
|  |  | R:TTGTCCTTGGCCATGTCTCC |
| MLOC_58438 | Nitrate transporter | F:GTCTCCATATCGCAGGCCAA |
|  |  | R:AGTGCCTTATACGTGCTGGG |
| MLOC_65110 | Nitrate transporter | F: TCGCCTACTTCGTACCAGGA |
|  |  | R: TCCATTCTTCTGCAGGCTCC |
| MLOC_24530 | AP2 domain containing protein | F: CATCTCTGCGACACGACAGT |
|  |  | R: TACTCCTGCGCAACCTCAAG |
| MLOC_74463 | Germin-like protein 2-1 | F: GTGAACAACGGTAAGGTGCC |
|  |  | R: GACCCAAAGACAGCATTGGC |
| MLOC_79223 | bZIP transcription factor superfamily protein | F: TTCGACATGGAGTATGCCCG |
|  |  | R:AGTCTTCGACGATGGTGCTG |
| MLOC_20326 | Abscisic acid responsive elements-binding factor 3 | F: GTATCCCTTCGACACCGTGA |
|  |  | R: ATAAGCCTGCTTCCTCTGGC |
| MLOC_77291 | Acetylglutamate kinase-like protein | F: AGGGTTGCTTCACGTGTTGT |
|  |  | R: AGTCCAGTGGCAGATCTTGT |
| MLOC_37710 | Methylthioribose kinase | F: GTCCAGGTTCAGAGAACGCA |
|  |  | R: CTGATGAGCGAGTGGGGAAT |
| MLOC_67158 | Heat shock factor protein 1 | F: TCACACGCGTCCACAGATAC |
|  |  | R: AGGGAAACGCTGATGTCCTG |
| MLOC_64811 | Tyrosine-sulfated glycopeptide receptor 1-like | F: TACAACTTCTACGGCGAGGC |
|  |  | R: ACAACGGTATCTGGCCCTTG |
| M36650.1 | GAPDH | F:AAGCATGAAGATACAGGGAGTGTG |
|  |  | R:AAATTTATTCTCGGAAGAGGTTGTACA |
| U34198 | *HvNRT2.1* | F:TCCGCGACAACCTAAACCTC |
|  |  | R:GCATCCATATCGAGGGCCAA |
