# Supplementary material for: Transcriptomic analysis reveals adaptive strategies to chronic low nitrogen in Tibetan wild barley
Source: BMC Plant Biol. 2019 Feb 11;19:68. doi: 10.1186/s12870-019-1668-3 (PMC6371475; doi:10.1186/s12870-019-1668-3)
Supplement: Supplementary file 10 [file 12870_2019_1668_MOESM1_ESM.docx]

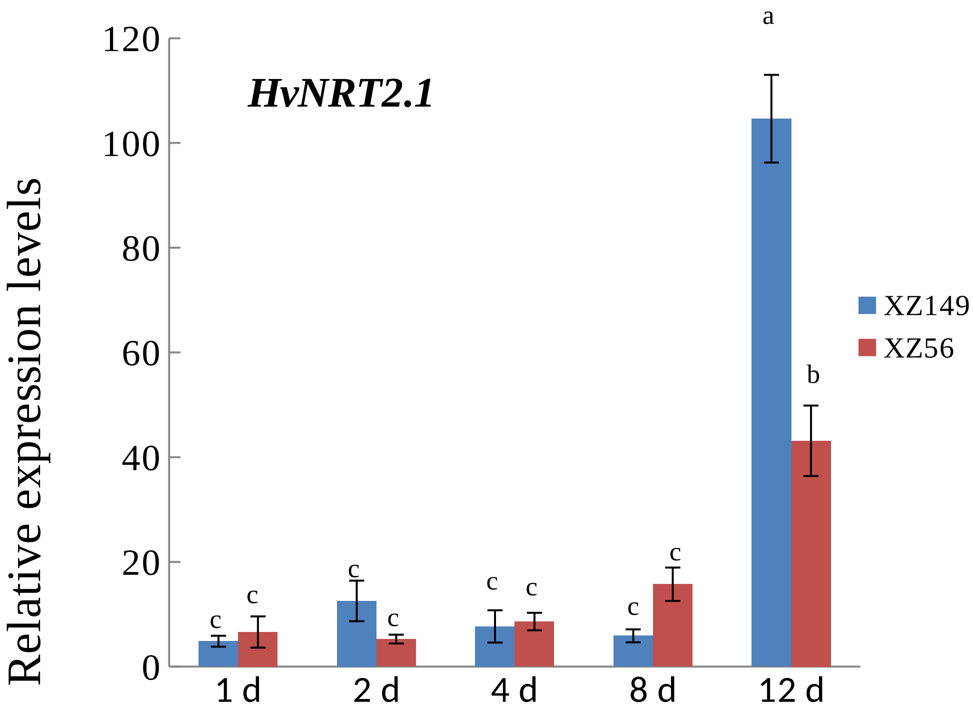

HvNRT2.1
Relative expression levels
120
100
80
60
40
20
0
1 d
2 d
4 d
8 d
12 d
XZ149
XZ56
a
b
c
